# A bibliometric analysis of rapid-eye-movement sleep behavior disorder

**DOI:** 10.3389/fneur.2026.1541715

**Published:** 2026-02-13

**Authors:** Mingyue Liu, Xuqian Liu, Wenyan Liu, Dandan Tian, Bin Zheng, Yinfan Xiao, Jing Zhang, Feng Xu, Wei Shang, Zhaohong Xie

**Affiliations:** 1 Department of Neurology, Second Hospital of Shandong University, Jinan, China; 2 Department of Neurology, Affiliated Hospital of Jining Medical University, Jining, China; 3 Department of Neurology, Linqing People’s Hospital, Liaocheng, China; 4 School of Management, Shandong University, Jinan, China

**Keywords:** bibliometrics, CiteSpace, Parkinson’s disease, RBD, REM sleep

## Abstract

Rapid-eye-movement sleep behavior disorder(RBD) is a parasomnia characterized by the loss of normal muscle atonia during rapid eye movement (REM) sleep, leading individuals to physically act out vivid, often intense or violent dreams, which may result in self-injury or harm to others. In recent years, RBD has become a prominent research focus. The bibliometrics can clearly and intuitively display the research profile, relationships, and clustering within the field. This study examines the development of RBD research using bibliometric analysis. As of 18 January 2026, articles on RBD in the Web of Science Core Collection (WoSCC) were collected. CiteSpace is utilized to analyze and visualize the attributes of the articles, including number of publications, institution, journals, authors, keywords, from the 4,229 publications obtained. The leading institution in this field was McGill University, and the most cited journal was *NEUROLOGY*. The most common keywords were “Parkinson’s disease” and “rem sleep behavior disorder.” The study conducted a bibliometric analysis of over 37 years of RBD research, identifying the authors, institutions, keywords, journals, and publications involved in this field. The findings provide a comprehensive overview of RBD research.

## 1 Introduction

Rapid-eye-movement sleep behavior disorder (RBD) is a parasomnia occurring during REM sleep, characterized by the loss of muscle atonia, accompanied by vigorous behaviors and abnormal muscle activity related to unpleasant dreams, which may cause harm (1, 2). It can be categorized into idiopathic RBD (iRBD) and symptomatic RBD (sRBD) (3). Idiopathic RBD (iRBD) is defined as RBD occurring in the absence of concurrent neurological diseases. It represents a prodromal stage of synucleinopathies, with the majority of cases progressing to Lewy body disorders (LBDs), such as Parkinson’s disease (PD) and dementia with Lewy bodies (DLB) (4, 5). And sRBD is associated with antidepressant use or with neurological diseases, especially *α*-synucleinopathies (such as PD, DLB, and multiple system atrophy) but also narcolepsy type 1 (6). Clinically, RBD presents with two key components: the loss of REM sleep atonia, evidenced by increased muscle tone during 8.7% to 100% of REM sleep, and the motor enactment of dreams, commonly manifesting as violent behaviors (kicking, punching, shouting, and verbal aggression) in response to perceived threats within the dream content (7). The diagnosis of RBD needs to be confirmed by laboratory sleep studies (polysomnograms) and video recordings, which help confirm abnormal behavior during REM sleep and rule out other sleep disorders. Treatment of RBD mainly involves reducing the risk of injury through behavioral changes and/or medication. According to current guidelines from the American Academy of Sleep Medicine (AASM), the recommended medication for iRBD in adults is fast-release melatonin, clonazepam, or pramipexole (8).

Bibliometrics, a branch of informatics, focuses on the literature system and its bibliometric characteristics as research objects, conducting both quantitative and qualitative analyses of literature. This approach enables quantitative measurement of the contour distribution, relationships, and clustering within a research field (9), and has emerged as one of the prevalent techniques for evaluating the credibility, quality, and impact of academic work (10–12). Specifically, such evaluations can cover the contributions and influence of different authors, countries/regions, institutions, disciplines, and journals, while also assessing the status, trends, and frontiers of research activities (13, 14). CiteSpace is commonly used bibliometric visualization tools for data analysis and visualization (15); it serves as an effective method for evaluating the thematic development of structured content and can enhance readers’ intuitive comprehension (16, 17).

While this type of literature analysis has been widely applied in other fields in recent years, to our knowledge, no bibliometric studies have been conducted in the field of RBD to date. To address this knowledge gap, the present study is based on the Web of Science™ Core Collection (WoSCC). Relevant bibliometric data (including annual number of articles, authors, institutions, journals, and keywords) in various RBD research fields were retrieved, and descriptive statistics were performed. This paper discusses the research status, hotspots, and frontiers of RBD-related literature, and uses CiteSpace to generate knowledge maps, aiming to provide a reference for future relevant research.

## 2 Materials and methods

### 2.1 Raw data acquisition

This study utilized the Web of Science (WOS) Core Collection database as the source of original literature, with the retrieval date set as January 18, 2026. The entire process of literature retrieval and screening strictly adhered to the Preferred Reporting Items for Systematic Reviews and Meta—Analyses (PRISMA) guidelines. The specific search strategy was formulated as (TS = “Rapid-eye-movement sleep behavior disorder” OR “REM sleep behavior disorder” OR “Rapid-eye-movement sleep behavior disorder” OR “REM sleep behavior disorder” OR “rem sleep behavior disorder” OR “rapid eye movement sleep behavior disorder”), yielding a total of 4,229 relevant literatures from WOS. All retrieved records, including full records and cited references, were exported in plain-text format.

The inclusion criteria were restricted to studies that: (1) incorporated the search terms; (2) involved fields related to RBD; (3) were published as research articles or reviews; and (4) were written in English. Additionally, the exclusion criteria were defined based on source types, including but not limited to meeting abstracts, editorial materials, corrections, letters, retractions, or conference proceedings.

**Figure 1:**
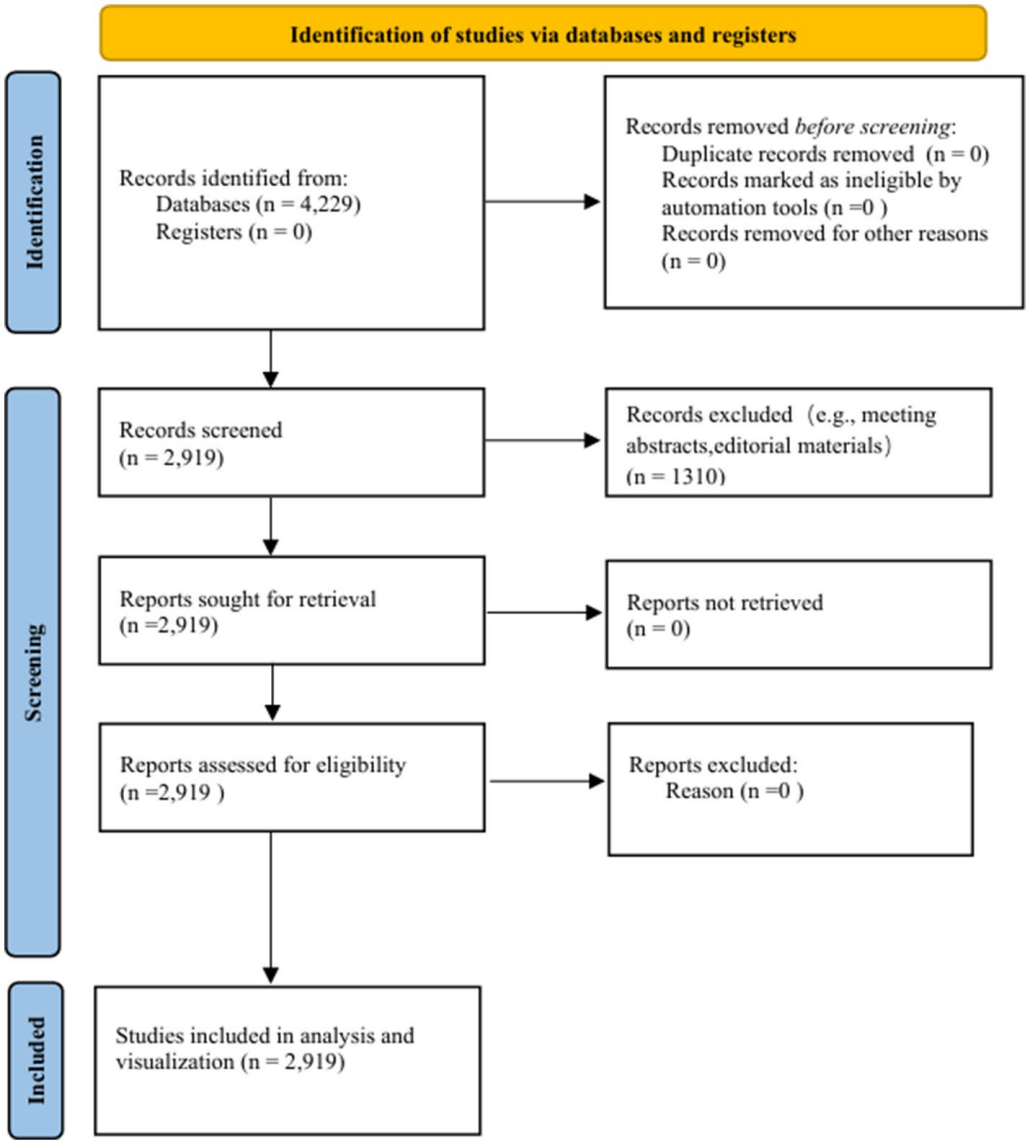
Flow-chart of the study. This flow-chart depicts the PRISMA-inspired study selection process, with key elements. Yellow top bar: represents initial study identification from databases/registers. Blue vertical bars: mark three phases (identification, screening, included), guiding record filtering. Arrows/boxes: Show step-by-step reduction of records (4,229→2,919), highlighting exclusions (duplicates, ineligible content) and final inclusion for analysis.

Upon completion of the initial search, two researchers independently screened and evaluated the literature to determine the relevance of each paper to the study’s focus. In cases of discrepancies, a third investigator was consulted to adjudicate. Using the aforementioned inclusion and exclusion criteria, a corpus of 2,919 studies was identified and subsequently downloaded for analysis and visualization. The detailed process from initial retrieval to final inclusion of literatures is clearly presented in the form of a flow chart (Figure 1), in line with the requirements of the PRISMA guidelines, to ensure the scientificity, standardization and repeatability of the research process.

### 2.2 Data analysis and related parameter settings

The data obtained from WoS was imported into Excel and CiteSpace software for analysis and statistics, with descriptions made on the number of publications, main authors, research institutions, keywords, and journal co-citation situations (Figure 1). By sorting out the studies related to RBD in recent years, the distribution structure and development trend of this field have been clarified.

In the operation of CiteSpace 6.2.R3 software, the time range is set from 1989 to 2026, the time slice is selected as 1 year, and the rest of the options were kept as default setting. The Pathfinder algorithm is used in the analysis of publishing institutions, author analysis, keyword analysis, keyword clustering, and journal co-citation analysis.

### 2.3 Analysis of visualization results

In the node type, institutions, journals, authors and keywords are analyzed separately. The graph generated by CiteSpace consists of nodes and the corresponding lines. The size of a node corresponds to the frequency of that node in the corresponding analysis module, the color of a node corresponds to the research time of that node, and the links between nodes indicate the co-occurrence or co-citation between nodes. In keyword detection, log-likelihood rate is usually used as the main method of keyword clustering analysis, and keyword class clusters with high confidence are clustered at the clustering module value (Modularity Q) and mean silhouette value (Mean Silhouette) based on co-occurrence analysis.

## 3 Results

### 3.1 Analysis of the number of articles issued

**Figure 2:**
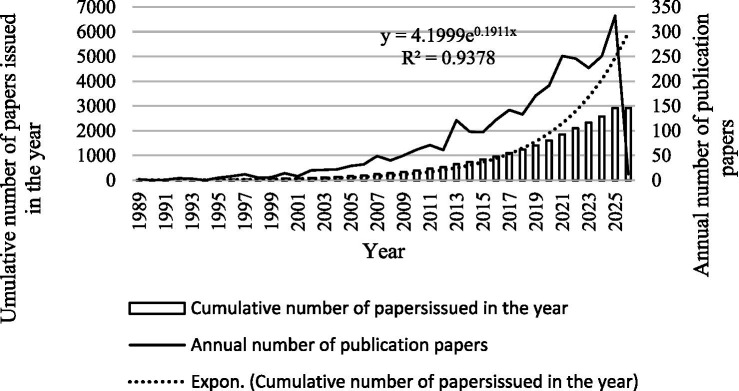
Annual changes in the number of publications related to RBD in Web of Science core collection (1989-January 2026).

A total of 4,229 papers were screened from the WoSCC database, among which 2,919 were included in this bibliometric study, consisting of 2,419 research articles and 500 reviews. Figure 2 clearly presents the annual publication volume and annual cumulative publication volume in this field. The annual publication volume reflects the development trend of this discipline, which is currently in a stage of rapid expansion.

The earliest relevant literature was published in 1989. We delineated the developmental trajectory of this research field into four distinct phases based on publication dynamics: (1) the Emergence and Preliminary Exploration Phase (1989–2010), during which annual publication output remained consistently below 50 papers, cumulative publications grew at a sluggish pace, and the field was still in its initial exploratory stage; (2) the Steady Growth Phase (2011–2018), where annual publications escalated gradually from 50 to approximately 150 papers, with cumulative publications entering a robust linear growth trajectory; (3) the Rapid Expansion Phase (2019–2022), in which annual publication volume surpassed the threshold of 200 papers and continued to climb steadily, while cumulative publications diverged from linear growth and shifted into an accelerated growth phase; (4) the Explosive Growth Phase (2023–2026), characterized by annual publications approaching 350 papers, cumulative publications exhibiting a prominent exponential growth pattern, a trend corroborated by a high goodness-of-fit index (*R*^2^ = 0.9378) from the exponential regression model.

To further predict the publication trend of RBD, an exponential function describing the annual publication trend in this field from 1989 to 2026 was selected based on the correlation coefficient R^2^. Analysis shows that the exponential function *y* = 4.1999e ^0.1911x^ (where *R*^2^ = 0.9378, y represents the annual number of publications, and x represents the year) indicates that the number of publications in RBD-related fields will continue to grow in the next few years.

### 3.2 Author collaboration map analysis

**Figure 3:**
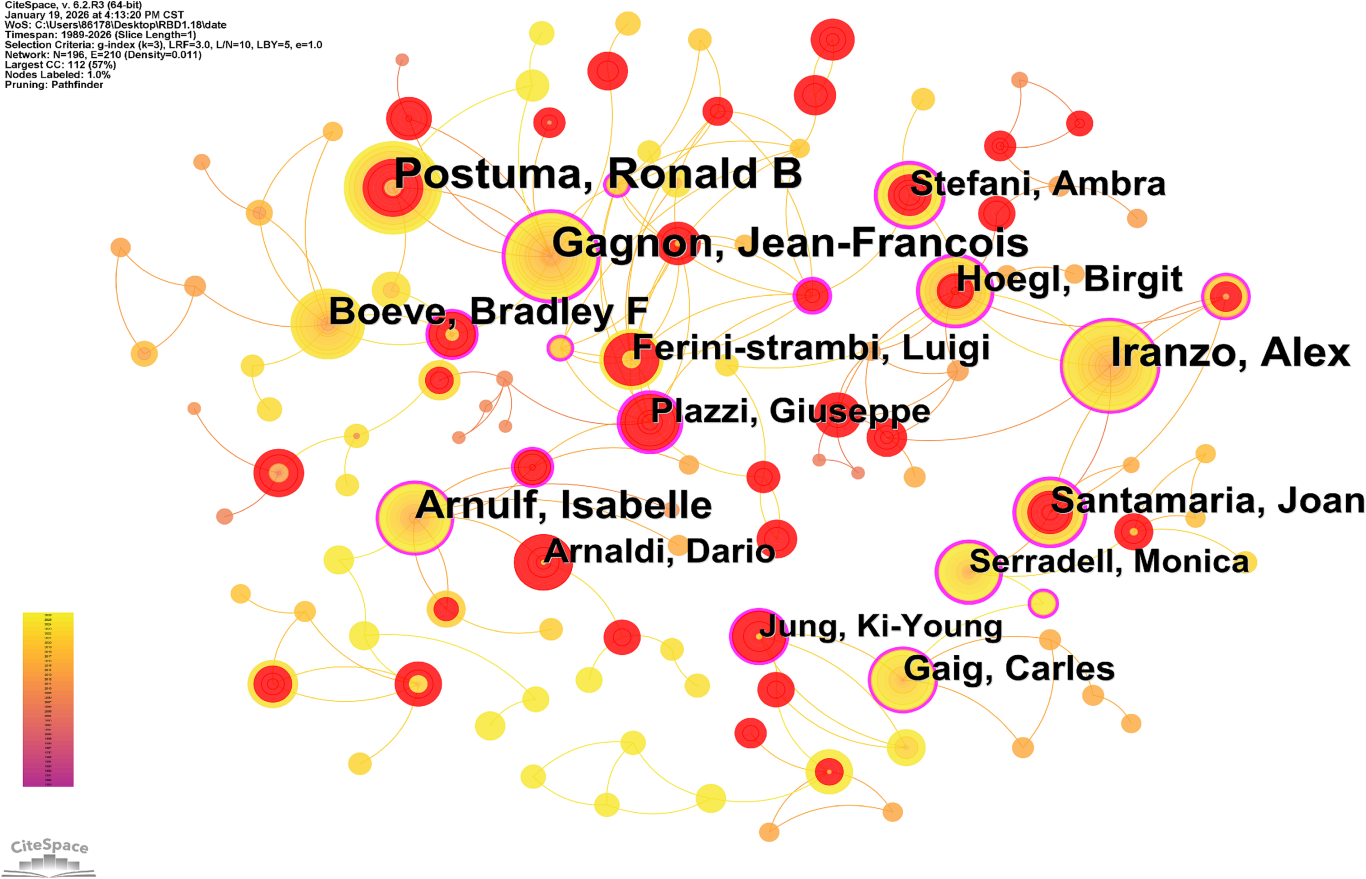
Author collaboration maps in RBD-related fields in the Web of Science core collection (1989-January 2026). Nodes represent authors, and the larger the node, the higher the number of papers. The lines represent the citations by other authors, and the thicker the line, the higher the citation frequency.

A total of 11,293 authors contributed to this research Table 1 presents the top 10 authors ranked by the number of published papers, with Ronald B Postuma, Alex Iranzo, and Jean-François Gagnon being the top three in this order. Ronald B Postuma is the most prolific author, with 123 publications to his credit, closely followed by Alex Iranzo, who has 107 papers. Figure 3 visualizes the author collaboration networks within this field. Notably, highly productive authors exhibit a pattern of close collaborative ties (18), among which a robust collaborative relationship has been established between Ronald B Postuma and Jean-François Gagnon.

**Table 1:** Top 10 authors with the most publications in RBD-related fields in the Web of Science core collection (1989-January 2026).

Number	Frequency	Centrality	Year	Author
1	123	0.01	2010	Postuma, Ronald B
2	107	0.12	2007	Iranzo, Alex
3	104	0.24	2006	Gagnon, Jean-Francois
4	82	0.19	2007	Arnulf, Isabelle
5	69	0.09	2006	Boeve, Bradley F
6	60	0.34	2010	Hoegl, Birgit
7	56	0.22	2007	Santamaria, Joan
8	52	0.03	2013	Ferini-strambi, Luigi
9	48	0.16	2015	Gaig, Carles
10	48	0.24	2020	Stefani, Ambra

### 3.3 Analysis of institution publications

**Figure 4:**
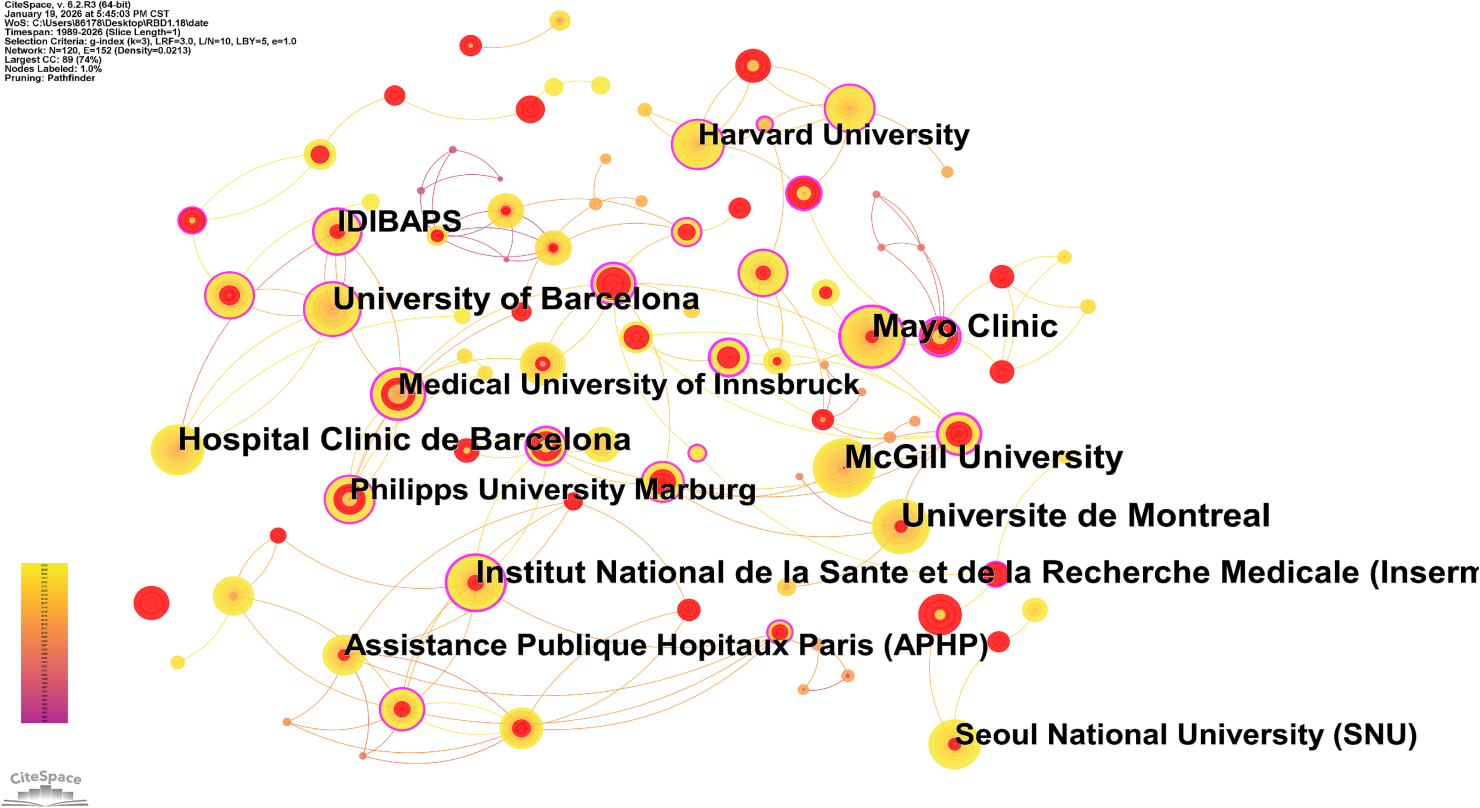
Institutions collaboration maps in RBD-related fields in the Web of Science core collection (1989-January 2026). Nodes represent institutions, and the larger the node, the higher the number of papers. The lines represent the citations by other institutions, and the thicker the line, the higher the citation frequency.

To explore institutions’ contributions to RBD, the number of publications from various institutions was analyzed. Globally, approximately 9,979 institutions have engaged in RBD-related research. Figure 4 intuitively presents the global institutional collaboration network and publication dynamics in the field of RBD research. As can be seen from the figure, various institutions are represented in the form of nodes, with red nodes highlighting institutions that have experienced rapid growth in publication volume recently. McGill University emerged as the leading institution in this field. Notably, the dense interconnections among IDIBAPS, the University of Barcelona, and Hospital Clinic de Barcelona form a robust regional collaborative network, while cross-regional partnerships (e.g., between the Mayo Clinic and McGill University) underscore the extensive scope of international cooperation in this research domain. At the same time, Table 2 also clearly marks the top 10 core institutions ranked by the number of publications, providing an intuitive reference for understanding the institutional distribution of global RBD research.

**Table 2:** Top 10 institutions with the most publications in RBD-related fields in the Web of Science core collection (1989-January 2026).

Number	Frequency	Centrality	Year	Institution
1	175	0	2006	McGill University
2	171	0.05	1992	Universite de Montreal
3	155	0.43	1999	Mayo Clinic
4	143	0.01	1999	Hospital Clinic de Barcelona
5	137	0.3	2007	Institut National de la Sante et de la Recherche Medicale (Inserm)
6	130	0.19	1999	University of Barcelona
7	106	0.24	2005	IDIBAPS
8	101	0	2005	Assistance Publique Hopitaux Paris (APHP)
9	97	0.05	2014	Seoul National University (SNU)
10	96	0.11	2013	Harvard University

### 3.4 Keyword co-occurrence analysis

**Figure 5:**
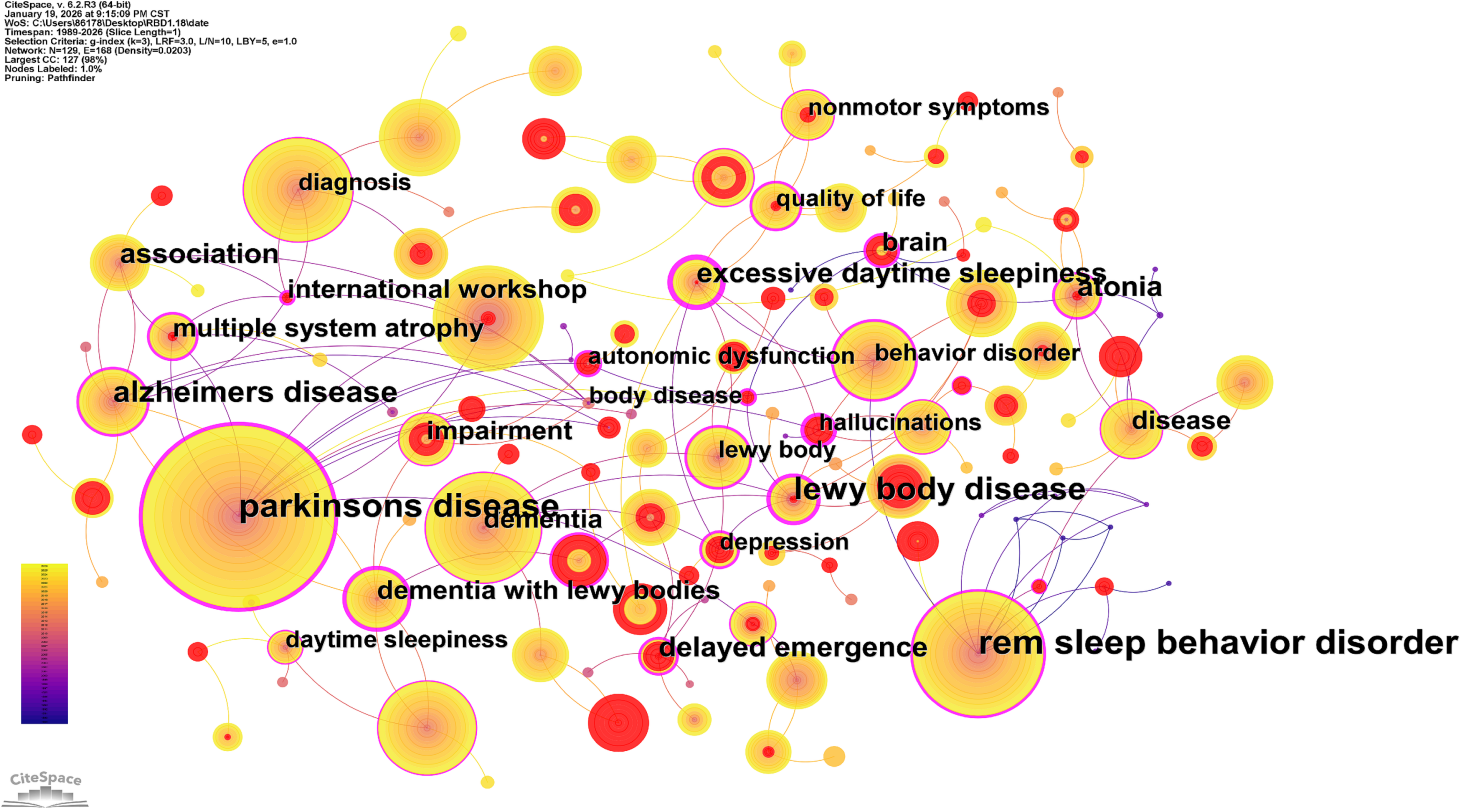
Keyword maps in RBD-related fields in the web of science core collection (1989-January 2026). Nodes represent keywords (larger nodes indicate a greater number of occurrences of the corresponding keyword).

Keywords represent the core content and research themes of the literature. Through keyword co-occurrence analysis it becomes feasible to comprehend the development and distribution of diverse research hotspots within a specific field (19). After screening and merging there were 129 nodes for keywords and 168 connections between nodes (Figure 5). Table 3 presents the 10 most frequently recorded keywords by authors and indexers. “Parkinson’s disease” stands as the most extensively studied keyword which underscores the core value of RBD as a prodromal marker for it (20). Furthermore there exist numerous keywords associated with RBD including “rem sleep behavior disorder” “sleep behavior disorder” “rem sleep” and “dementia.”

**Table 3:** Top 10 ranking of keywords in RBD-related fields in the Web of Science core collection (1989-January 2026).

Number	Frequency	Centrality	Year	Keyword
1	1,480	0.54	1998	Parkinson’s disease
2	792	0.37	1991	Rem sleep behavior disorder
3	574	0.19	2001	Dementia
4	563	0	1995	Rem sleep
5	492	0.18	2002	Diagnosis
6	400	0.18	2004	Sleep behavior disorder
7	337	0.39	1995	Behavior disorder
8	276	0.06	2010	Risk
9	242	0.28	1997	Alzheimer’s disease
10	205	0	2010	Mild cognitive impairment

### 3.5 Keyword clustering analysis

**Figure 6:**
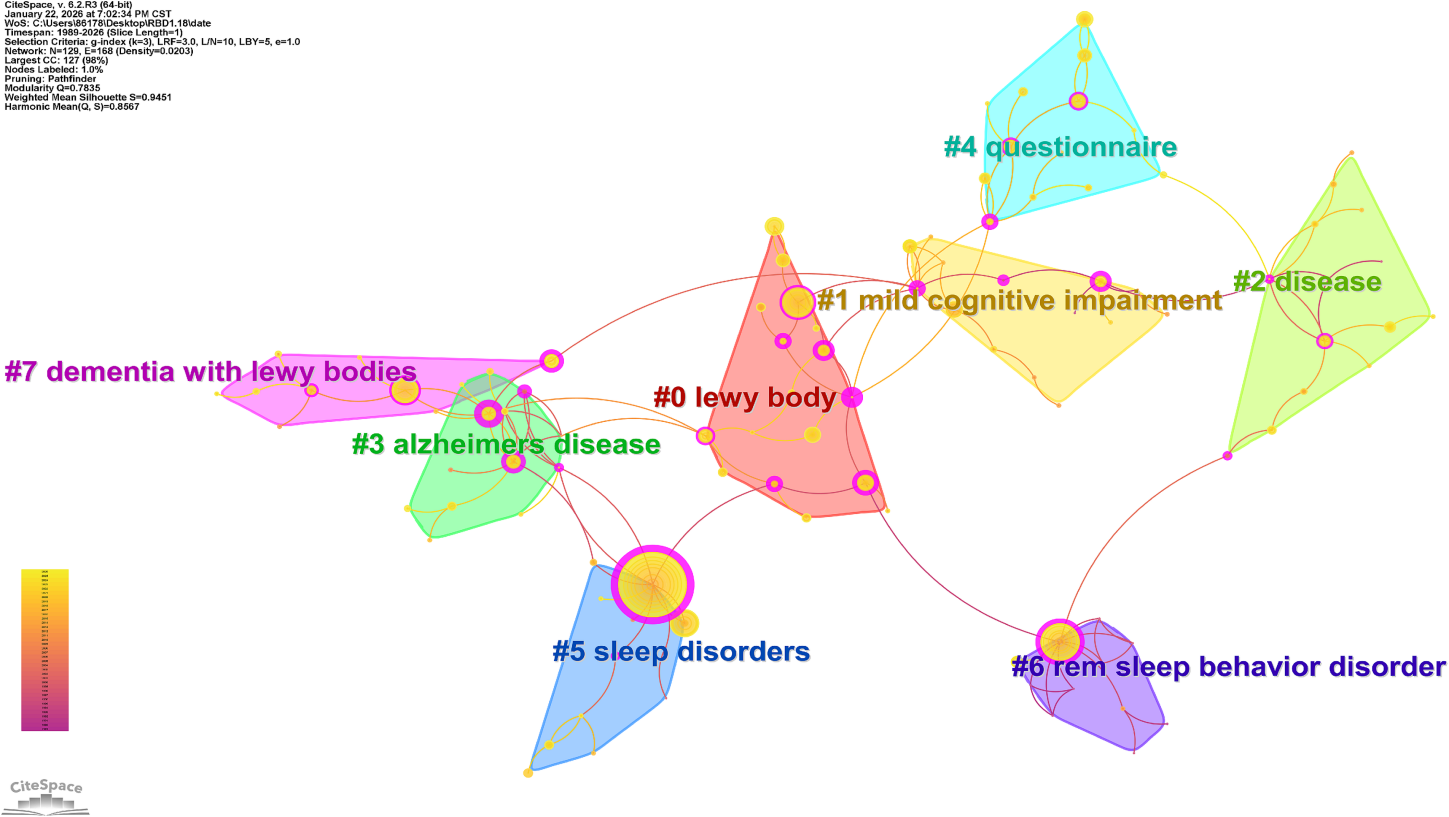
Keyword clustering maps in RBD-related fields in the Web of Science core collection (1989-January 2026). Color represents clustering, and nodes with the same color belong to the same cluster.

Cluster analysis identifies components of keywords with similarities and groups them into clusters, thereby illustrating the current research landscape of the field (21). In Figure 6, Modularity *Q* = 0.8475 > 0.3 and Weighted Mean Silhouette S = 0.9653 > 0.7 indicate that this structure of clustered associations is significant and effective. A total of 8 clusters are identified in the keyword clustering map (Figure 6), specifically: “lewy body,” “mild cognitive impairment,” “disease,” “Alzheimer’s disease,” “questionnaire,” “sleep disorders,” “rem sleep behavior disorder,” “dementia with lewy bodies.” These eight keyword clusters could be thematically categorized into four distinct groups, each representing a core research dimension of the field: core pathological substrates and disease entities, which encompasses Lewy bodies, dementia with Lewy bodies and Alzheimer’s disease, and underscores the pathological overlap among these disorders and their differential diagnosis as the fundamental research focus of this field (22); the cognitive impairment continuum, which includes mild cognitive impairment and broader neurodegenerative disease phenotypes, and highlights the progressive trajectory of cognitive decline as a cornerstone for clinical diagnosis and disease monitoring (23); sleep-related comorbidities and prodromal markers, which consists of sleep disorders and REM sleep behavior disorder, and reflects the well-recognized role of sleep disturbances as critical early prodromal indicators and common concomitant features of Lewy body diseases, with profound implications for disease risk stratification and early intervention; and clinical assessment instruments, represented by questionnaires, which underscores the indispensable reliance on standardized evaluation tools for systematic symptom assessment and longitudinal data collection, thereby ensuring the methodological rigor of clinical research and practice in this domain.

### 3.6 Journal co-citation analysis

**Figure 7:**
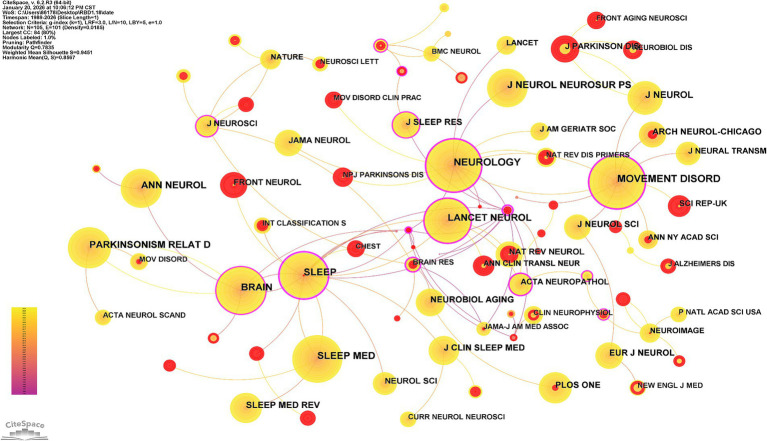
Co-citation journal collaboration maps in RBD-related fields in the Web of Science core collection (1989-January 2026). Nodes represent journals (larger nodes indicate higher citations of RBD-related papers published by the journal); lines represent journal co-citation links (thicker lines indicate higher co-citation frequency between the linked journals).

The original literature data obtained was imported into CiteSpace for analysis, and a journal co-citation map was generated to detect and evaluate influential journals that contribute to RBD research and development, acting as a knowledge base to a certain extent, as shown in Figure 7. A co-cited journal is one in which two or more publications in the field of RBD cite papers in the same journal. The CiteSpace configuration is set as follows: TopN (*N* = 20), LRF = 3, LBY = 5, e = 1.0, and the sliced and merged network is cut, resulting in 105 nodes and 101 connections.

Table 4 presents the top 10 most co-cited journals in the RBD field. *NEUROLOGY* was cited the most, 2,555 times. Of the top 10 journals totaling citations in the field of RBD, the journal with the highest current impact factor is *LANCET NEUROL* (IF = 45.5), followed by *BRAIN* (IF = 11.7). Impact factor and JCR are commonly used indicators to evaluate the influence of journals. JCR divides all journals into four quartiles (Q1–Q4) based on their IF (24). In the top 10 journals by paper count, Q1 journals account for 80%.

**Table 4:** Top 10 ranking of journal co-citations in RBD-related fields in the Web of Science core collection (1989-January 2026).

Number	Journals	Number of co-citations	Centrality	IF	JCR-C
1	Neurology	2,555	0.59	8.5	1
2	Movement Disord	2,392	0.42	7.4	1
3	Brain	2077	0.18	11.7	1
4	Sleep	1981	0.76	4.9	1
5	Sleep Med	1889	0.06	3.4	2
6	Lancet Neurol	1,641	0.3	45.5	1
7	Ann Neurol	1,603	0.03	7.7	1
8	J Neurol Neurosur PS	1,565	0	7.5	1
9	Parkinsonism Relat D	1,431	0.06	3.4	2
10	J Neurol	1,113	0.09	4.6	1

## 4 Discussion

This bibliometric study systematically analyzed publication trends, collaborative networks, and research hotspots in the field of RBD from 1989 to 2026, with data retrieved from the WoSCC. Our findings comprehensively delineate the developmental trajectory and core research landscape of RBD, thereby providing a robust framework for researchers to contextualize their investigations and identify future research priorities.

### 4.1 Staged developmental dynamics and underlying drivers

The temporal evolution of RBD-related publications exhibits a distinct four-phase pattern, which mirrors the progressive advancement of clinical and scientific understanding in this domain. During the Emergence and Preliminary Exploration Phase (1989–2010), annual publication output remained consistently below 50 papers, a reflection of the nascent stage of RBD research characterized by limited consensus on diagnostic criteria and a paucity of mechanistic insights. The Steady Growth Phase (2011–2018) witnessed a gradual rise in annual publications from 50 to approximately 150, a trend driven by the establishment of standardized diagnostic frameworks—most notably the *International Classification of Sleep Disorders, Third Edition (ICSD-3)*—which enabled rigorous multicenter investigations. The Rapid Expansion Phase (2019–2022) and Explosive Growth Phase (2023–2026) were distinguished by annual publication outputs exceeding 200 and approaching 350 papers, respectively. This accelerated growth was underpinned by the definitive recognition of RBD as a prodromal marker for Lewy body diseases, alongside technological breakthroughs in neuroimaging and molecular pathology (25). Our exponential regression model (*R*^2^ = 0.9378) validates this sustained growth trajectory, forecasting that RBD research will remain a high-priority research area aligned with the global imperative for the early intervention of neurodegenerative diseases.

### 4.2 Core research leadership and global collaborative architecture

Author and institutional collaboration analysis identify the key contributors and structural collaborative patterns shaping the landscape of RBD research (26). Ronald B Postuma, Alex Iranzo, and Jean-François Gagnon emerge as the most prolific authors in the field; notably, Postuma and Gagnon have forged a particularly impactful and enduring collaborative partnership that has advanced the development of RBD diagnostic algorithms and risk stratification models (27, 28). At the institutional level, the dense collaborative network formed by the IDIBAPS, the University of Barcelona, and Hospital Clinic de Barcelona exemplifies the strengths of regional academic clusters in integrating clinical resources and driving translational RBD research. Concurrently, interregional collaborations—such as that between the Mayo Clinic and McGill University—underscore the globalization of RBD research endeavors, enabling large-scale, multi-cohort studies that enhance the generalizability of research findings. This dual model of regional academic excellence and international research partnership is critical to accelerating scientific progress in the field (29).

### 4.3 Research hotspots and thematic dimensions

Keyword co-occurrence and clustering analysis—validated by a high modularity index (Q = 0.8475) and weighted mean silhouette value (S = 0.9653)—reveal four interconnected thematic pillars of RBD research with Parkinson’s disease emerging as the most frequently co-occurring keyword which corroborates the central role of RBD as a prodromal marker given that over 80% of idiopathic RBD patients progress to Lewy body diseases (30). Specifically the first pillar core pathological substrates and disease entities is illustrated by the clustering of Lewy bodies dementia with Lewy bodies and Alzheimer’s disease highlighting the pathological overlap among these conditions and identifying this as a critical focus for optimizing differential diagnosis and elucidating disease heterogeneity (22, 31); the second pillar the cognitive impairment continuum underscores the clinical significance of monitoring cognitive decline in RBD patients through the inclusion of mild cognitive impairment as cognitive dysfunction serves as both an early clinical manifestation and a key prognostic indicator in this cohort; the third pillar sleep-related comorbidities and prodromal markers—the electrophysiological gold standard for RBD diagnosis—alongside broader sleep disorders reinforcing the dual role of sleep disturbances as diagnostic criteria and early biomarkers for neurodegeneration (32, 33); and the fourth pillar clinical assessment instruments recognizes questionnaires such as the REM sleep behavior disorder screening questionnaire (RBD-SQ) as indispensable tools for large-scale epidemiological screening with the integration of these subjective assessments with polysomnography ensuring methodological rigor in both clinical practice and research contexts (34, 35).

### 4.4 Journal co-citation landscape and academic influence

Journal co-citation analysis identifies the primary knowledge dissemination channels in the field of RBD research. *Neurology* ranks first with 2,555 co-citations, affirming its status as the premier publication outlet for clinical RBD studies, while *Lancet Neurology* (IF = 45.5) and *Brain* (IF = 11.7) lead in publishing high-impact mechanistic research. Notably, 80% of the top 10 most co-cited journals fall into the JCR Q1 quartile, underscoring the high academic quality and international visibility of RBD scholarship. Beyond disseminating research findings, these core journals also shape the field’s research agenda through commissioned reviews and editorial perspectives, thereby driving collective scientific progress in RBD research (36).

### 4.5 Future directions

Moving forward, future research should prioritize four key directions: integrating multi-omics and advanced neuroimaging technologies to identify subtype-specific biomarkers of RBD progression; conducting large-scale randomized controlled trials to validate disease-modifying interventions for prodromal Lewy body diseases; developing artificial intelligence (AI)-driven tools for automated RBD diagnosis and risk stratification; and expanding global collaborative networks to include underrepresented populations, thereby enhancing the generalizability of research findings.

In conclusion, RBD research has entered an era of explosive growth, fueled by its well-established role as a prodromal marker for Lewy body diseases. As the field continues to mature, a sustained focus on mechanistic clarity, translational impact, and global collaborative efforts will be instrumental in unlocking RBD’s full potential—ultimately paving the way for improved early diagnosis and targeted treatment of neurodegenerative disorders.

## 5 Limitations

This study has several limitations. First, the data were derived solely from the WoSCC database, potentially excluding relevant literature from other platforms (e.g., Scopus or PubMed Central). Second, keyword clustering and co-occurrence analyses are dependent on the accuracy of indexing, which may introduce bias. Third, the ambiguity in paper classification, particularly in interdisciplinary journals, can be regarded as a methodological limitation of the bibliometric review itself, which may affect the accuracy of the analysis and should be included as a limitation. We confirm this ambiguity is an inherent limitation of this study. RBD literatures are often published in interdisciplinary journals, but common bibliometric classification frameworks have a “single-discipline orientation,” simplifying the interdisciplinary nature of RBD papers and causing inaccuracies in relevant indicators, thus affecting analysis accuracy. Fourth, this study did not address “journal nesting.” Undecomposed nesting may overstate citation counts (e.g., lumping high-cited papers into one category distorts results). Future studies could improve this by classifying via unique ISSN or building a publisher-journal correspondence table.

## 6 Conclusion

In summary, this bibliometric study systematically analyzed the global research landscape of RBD from 1989 to 2026 using WoSCC data. The findings confirm that RBD research has entered an explosive growth stage, primarily driven by its well-recognized role as a key prodromal marker for Lewy body diseases. An exponential regression model (*R*^2^ = 0.9378) validates this sustained upward trend, which has evolved through four distinct developmental phases from initial exploration to explosive growth. The leading institution in this field was McGill University. And the field adopts a dual collaborative model, integrating regional academic clusters and global interregional partnerships, which effectively promotes research progress and result generalizability. Keyword co-occurrence and clustering analysis identify four core research pillars centered on Parkinson’s disease, highlighting RBD’s critical value in the early diagnosis and intervention of neurodegenerative diseases. Journal co-citation analysis shows that high-impact journals such as *Neurology* and *Lancet Neurology* ensure the field’s high academic quality and international influence. This study provides a comprehensive framework for understanding the current status and research hotspots of RBD. Future research should prioritize mechanistic exploration of RBD progression, validation of disease-modifying interventions *via* large-scale clinical trials, development of AI-driven diagnostic tools, and expansion of global collaboration to further unlock RBD’s potential in the early diagnosis and targeted treatment of neurodegenerative diseases.

## Data Availability

The raw data supporting the conclusions of this article will be made available by the authors, without undue reservation.
